# Risk of Coronary Heart Disease among HIV-Infected Patients: A Multicenter Study in Brazil

**DOI:** 10.1155/2013/163418

**Published:** 2013-10-02

**Authors:** Sandra C. Fuchs, Paulo R. Alencastro, Maria Letícia R. Ikeda, Nêmora T. Barcellos, Fernando H. Wolff, Ajácio B. M. Brandão, Ricardo A. A. Ximenes, Demócrito de B. Miranda-Filho, Heloísa Ramos Lacerda, Maria de Fátima P. M. de Albuquerque, Ulisses Ramos Montarroyos, Max W. Nery, Marilia D. Turchi

**Affiliations:** ^1^Postgraduate Studies Program in Cardiology, School of Medicine, Clinical Research Center, No. 5 Andar, Hospital de Clinicas de Porto Alegre, Universidade Federal do Rio Grande do Sul, Rua Ramiro Barcelos 2350, 90035-903 Porto Alegre, RS, Brazil; ^2^Postgraduate Studies Program in Epidemiology, School of Medicine, Universidade Federal do Rio Grande do Sul, 90035-003 Porto Alegre, RS, Brazil; ^3^National Institute for Health Technology Assessment (IATS/CNPq), Hospital de Clinicas de Porto Alegre, 90035-903 Porto Alegre, RS, Brazil; ^4^Department of Clinical Medicine, Universidade de Pernambuco, 50670-901 Recife, PE, Brazil; ^5^Department of Tropical Medicine, Universidade Federal de Pernambuco, 50610-110 Recife, PE, Brazil; ^6^Research Center Aggeu Magalhães, Fundação Oswaldo Cruz, 50.670-420 Recife, PE, Brazil; ^7^Institute of Biological Sciences, Universidade de Pernambuco, 50670-901 Recife, PE, Brazil; ^8^Institute of Tropical Pathology and Public Health, Universidade Federal de Goiás, 74605050 Goiania, GO, Brazil

## Abstract

Cardiovascular disease has emerged as a crescent problem among HIV-infected population. This study aimed to determine the 10-year risk of coronary heart disease using the Framingham risk score among HIV-infected patients from three regions of Brazil. This is a pooled analysis of three cohort studies, which enrolled 3,829 individuals, 59% were men, 66% had white skin color, and mean age 39.0 ± 9.9 years. Comparisons among regions showed that there were marked differences in demographic, socioeconomic, clinical, and HIV-related characteristics. Prevalence of Framingham score ≥10 was 4.5% in the Southern, 4.2% in the Midwest, and 3.9% in the Northeast of Brazil. The Framingham score ≥10 was similar between regions for males, patients aged ≥60 years, with obesity, central obesity, hypertension, and diabetes mellitus. Women were three times more likely to have coronary heart disease in 10 years than men. Hypertension and diabetes increased more than four times the risk of coronary heart disease, followed by central obesity, obesity, and prehypertension. The use of antiretroviral agents and time since HIV diagnosis were not risk factors for coronary artery disease in 10 years. In conclusion, hypertension and diabetes are the strongest independent predictors of 10-year risk of coronary heart disease among HIV-infected population.

## 1. Introduction 

Cardiovascular disease (CVD) has emerged as a growing problem among HIV-infected population. With the advent of highly active antiretroviral therapy (HAART), there was a reduction in AIDS-related mortality, increasing of life expectancy, and the exposure to traditional cardiovascular risk factors [1–3]. On the other hand, the infection itself, as well as HAART, seems to be involved in changing the profile of cardiovascular risk factors [4, 5]. Dyslipidemia and hyperglycemia are adverse effects of HAART, which were associated with metabolic syndrome and are intermediate steps in the development of cardiovascular events [6, 7].

In Brazil, the use of antiretroviral therapy (ART) is available to the public free of charge, as well as blood pressure-lowering agents, and other medications to control risk factors and prevent CVD. However, there are limited data on coronary heart disease (CHD) in the HIV-infected population [8, 9]. Cardiovascular risk can be evaluated by means of equations that combine several risk factors to provide a quantitative estimate of the risk [10]. The Framingham risk equation has been widely used to estimate the risk of development coronary heart disease over a fixed period of time, usually 10 years, in the general population [11], but the information in the HIV-infected population need to be further addressed [12]. 

Since the creation of the original equation [11], the Framingham risk score has been modified [13], and some concerns about its use were raised [14]. Although it has been suggested that a specific score needs to be used [15, 16], it is still uncertain the magnitude of CHD burden in the HIV-infected population. Moreover, the diversity of exposure to risk factors and socioeconomic conditions among patients from different clinical settings in Brazil may require the inclusion of different components in the score. Therefore, this study aimed to determine the 10-year risk of coronary heart disease using the Framingham risk score in HIV-infected patients from three regions of Brazil. 

## 2. Methods 

This is a cross-sectional with joint database analysis of baseline characteristics of three cohort studies, conducted in the Northeast, Midwest, and Southern Brazil, addressing characteristics associated with the Framingham risk score among HIV-infected individuals. In Recife, capital of the state of Pernambuco (Northeast), HIV-infected patients, aged 17 to 74 years, seen in two of the largest public outpatient centers (Hospital Universitario Oswaldo Cruz, from Universidade de Pernambuco, and Hospital Estadual Correia Picanço, from Health Secretariat of the state) for HIV/AIDS were enrolled. In Goiania, capital of the state of Goias (Midwest), HIV-infected patients, attending an outpatient public referral center for infectious diseases (Hospital das Clinicas da Universidade Federal de Goias), aged 20 to 75 years, with no clinical evidence of active opportunistic diseases at enrollment, were eligible to participate. In Porto Alegre, capital of the state of Rio Grande do Sul (Southern Brazil), HIV-infected patients aged 18 years or older, who have been seen in the outpatient clinic of the Hospital Sanatorio Partenon (SAT), of the Health State Department, were enrolled. In all studies, pregnant women, patients with mental retardation, and under restriction of freedom were excluded. The data collection was conducted in 2007–2009 (Recife), 2009–2011 (Goiania), and 2006–2008 (Porto Alegre). All research projects have been approved by the Institutional Review Board of the institutions, which are accredited by the Office of Human Research Protections, and all participants signed an informed consent. 

Patients were interviewed using similar standardized questionnaires, anthropometric and blood pressure measurements, while HIV-related variables were obtained from medical records. Data collection was performed on routine visits, by certified physicians and research assistants, using similar questions pertaining demographic characteristics (age, categorized as <50, 50–59, or ≥60 years; self-reported skin color, categorized as white or nonwhite), socioeconomic level (number of years of formal education completed successfully, further categorized as ≥10 or <10), lifestyle (physical activity, categorized as yes or no; lifetime smoking, and binge drinking), clinical characteristics (self-reported previous morbidity, blood pressure lowering agents, antidiabetic agents, and drugs used in the dyslipidemia treatment), HIV-related characteristics (current or past cocaine or crack cocaine use, categorized as yes or no; reported use of antiretroviral medicine, also confirmed by medical records, and duration of HIV infection since the date of diagnosis, categorized as <8 or 8–29 years), and other risk factors for coronary heart disease (body mass index, waist circumference, diabetes mellitus, and hypertension status). In all studies, smoking was investigated by several questions: current status, number of cigarettes smoked per day, and smoking cessation, which allowed classifying individuals as never, former or current smokers. In two studies (in the Northeast and Southern Brazil), these questions were asked to those who reported having smoked at least 100 cigarettes during lifetime [17]. Alcohol consumption was investigated using a standardized questionnaire [18], including type, quantity, and frequency of each beverage consumed and binge drinking, defined by a consumption of five or more drinks on a single occasion [19].

Weight and height were obtained to calculate body mass index (kg/m^2^), classified as <25, 25–29, or ≥30 kg/m^2^. Central obesity was determined by waist circumference, which was measured in duplicate and classified as ≥102/88 cm for men and women, respectively. In Porto Alegre, there were four standardized measurements of blood pressure in two visits, using oscillometric monitor OMRON CP-705, and blood pressure was classified based on the average. In Recife and Goiania, there were two and three, respectively, measurements of blood pressure using calibrated aneroid sphygmomanometer (WelchAllyn/Tycos), and the average was used to classify blood pressure on <120/80, 80–89 and 120–139, or ≥140/90 mmHg, or use of antihypertensive medication. Laboratory tests were performed on fasting blood samples, with three months around the date of the interview. Diabetes mellitus was diagnosed based on fasting glucose ≥126 or use of antidiabetic agents. 

Framingham scoring was calculated based on age, sex, total cholesterol, HDL cholesterol, LDL cholesterol, smoking status, diabetes mellitus, and blood pressure [11, 20], besides the reported use of using blood pressure, diabetes, or cholesterol lowering agents. Estimates of the Framingham score are more robust for total cholesterol than for LDL-cholesterol [21], and since LDL is the main treatment target [13], the lipid profile (total cholesterol, HDL, and LDL cholesterol) was maintained in the score calculation. The total score was calculated based on the original score sheets, which provide risk of CHD compared to people of the same age and sex [11]. The 10-year risk of CHD score was categorized as <10 (low) or ≥10 (intermediate or high risk, due to the expected low number of participants with high scores) to calculate prevalence and independent risk ratios with 95%CI (confidence intervals). This cutoff was based on the recommendations of the Adult Treatment Panel III (ATP III), which identified categories of cardiovascular risk to determine goals for lipid-lowering therapy [13]. 

### 2.1. Statistical Analysis

A conceptual model through a hierarchical procedure was adopted in the data analysis in order to take confounding factors into account. The construction of the hierarchical model was based in two levels, and details are provided elsewhere [22]. Briefly, independent variables were grouped into socioeconomic (education), demographic factors (sex, age, and skin color), lifestyle (smoking, cocaine use, binge drinking, and physical activity), HIV characteristics (ARV use, length of HIV diagnosis) leading to direct determinants (obesity, hypertension, and diabetes mellitus) of cardiovascular disease [23]. Characteristics of the samples are expected to vary by regions, so an additional variable—the study site—was included in the multivariate analysis. At each hierarchical level, one regression equation was fitted including confounding factors which have been associated with Framingham score in the bivariate analysis (*P* level <0.2) or based on the literature. At the first level, the risk ratios (95% CI) were adjusted for the study site, sex, age, years at school, smoking, cocaine use, binge drinking, antiretroviral therapy, and years of the HIV diagnosis, and, at the second level, there was additional control for waist circumference, hypertension status, and diabetes mellitus. Multivariate analysis was performed using modified Poisson regression (robust variance estimates) [24], and analysis of prevalence of risk factors by regions was carried out using the chi-squared test, in the Statistical Package for Social Sciences (SPSS Inc., version 18, Chicago, IL, USA). 

Statistical power was calculated to assess the size of odds ratios that could be detected in this joint analysis using the Epidata statistical software (version 3.1, Pan-American Health Organization, Washington D.C., USA). The association between Framingham risk score ≥10 among participants with prehypertension versus normal blood pressure, for instance, with a 1.27 ratio of unexposed to exposed, significance level of 0.05 (two tailed), and 80% of statistical power would require a sample size of 1284 unexposed and 1011 prehypertensive participants to detect a risk ratio of 2.5.

## 3. Results

Table 1 shows the characteristics of 3,829 men (59.1%) and women (40.9%) evaluated in this pooled analysis of patients infected with HIV. Most were white (66%) had, on average, 39.0 ± 9.9 years, ranging from 18 to 84 years, but only 1.8% were older than 60 years and 40% finished 10 years or more in school. Comparisons among regions showed that there were marked differences in demographic, socioeconomic, clinical, and HIV-related characteristics. Compared with smoking prevalence detected in Southern Brazil, in the Midwest was about twice and in the Northeast three times more prevalent. In the Midwest, the prevalence of central obesity was about half of the prevalence identified in the Northeast and Southern Brazil.

The prevalence of Framingham score ≥10 was 4.5% in the Southern, 4.2% in the Midwest, and 3.9% in the Northeast of Brazil. The prevalence of intermediate or high Framingham score was similar between regions for males, patients aged ≥60 years, with obesity, central obesity, hypertension, and diabetes mellitus (Table 2). The association between alcohol abuse and intermediate or high Framingham score was detected only in the Northeast, while cocaine use was not associated with risk of CHD in Midwestern.

Results of the multivariate analysis showed that age over 50 years was an independent risk factor for coronary heart disease in 10 years (Table 3). Women were three times more likely to have coronary heart disease than men, regardless of age, socioeconomic status, lifestyle, and characteristics related to HIV. Age was the strongest predictor of CHD, even after the control for confounding factors. Binge drinking reduced CHD prevalence, independently of confounding factors, but there was a borderline significance. Hypertension and diabetes mellitus increased more than four times the risk of coronary heart disease, compared with the absence of these conditions, even after adjustment for confounding factors. Hypertension and diabetes were the strongest predictors of intermediate or high CHD risk, followed by central obesity, obesity, and prehypertension. The use of antiretroviral agents and time since HIV diagnosis were not independent risk factors for coronary artery disease in 10 years. Since most of risk factors are established at the age of 60 years, a subanalysis was carried out among participants younger than 50 years. Most of the associations between risk factors and intermediate or high Framingham score were also detected for those less than 50 years old, but some of the associations had even greater risk estimates, such as BMI, hypertension, and diabetes (data not shown). Independent of other confounding factors, an annual increase of age elevated by 30% a 10-year risk of an intermediate or high score (RR = 1.3, 95% CI: 1.2–1.4).

## 4. Discussion

The main objective of this study was to evaluate the association of risk factors and coronary heart disease in 10 years among HIV-infected patients from three regions of Brazil. Traditional risk factors such as age, hypertension, and diabetes mellitus were confirmed as independent risk factors, while HIV-related characteristics were not independently associated. Prehypertension was also independently associated with risk of CHD, which has been described in subjects not infected with HIV. Furthermore, the prevalence of risk factors by regions showed marked differences, suggesting that HIV-infected individuals living in several parts of Brazil did not share many characteristics besides infection.

These findings were not surprising, since age is a strong predictor of CHD risk and one of the main drivers of the Framingham score [20], while hypertension is prevalent and has accounted for almost half of ischemic heart disease cases worldwide [25]. Diabetes mellitus has been pointed out as an important risk factor for CHD [26] in HIV-infected individuals, but the risk detected in this pooled analysis was higher than previously described [27]. Among HIV-positive residents near Kampala, Uganda, in Africa, it was found that more than a quarter had hypertension, similar to the overall prevalence detected in this study, but it was assumed that no participant was smoker. Even so, there was an excessively high prevalence of Framingham score above 10% among men (42% versus 3.7% of women) [28]. In another study, conducted in Germany, about half of HIV-infected participants were smokers and a fifth had high blood pressure. However, approximately 22% and 18% of patients, respectively, were categorized as being at moderate or high 10-year risk for CHD [29]. Results of theoretical models [1, 30] and based on clinical data suggest an increased risk of myocardial infarction, due to the use of antiretroviral treatment. However, these results were found to be specific antiretroviral agents such as indinavir, lopinavir, ritonavir, didanosine, and abacavir [30–32]. In Brazil, most of these agents has been withdrawn or has not been employed in the public health system, and two non-NRTI agents, out of seven, were associated with risk of myocardial infarction. 

In this study, prevalence of high 10-year CHD risk (>10%) was very low, as previously reported in Brazil [12, 33]. Among potential explanations, one is that the HIV-infected population was under 60 years old and had low prevalence of cardiovascular risk factors. Furthermore, the Framingham risk score may not be the best tool for assessing cardiovascular risk [34]. Furthermore, it was developed in a subsample of the American population—middle-aged, mostly Caucasians—free of CHD at baseline, whereas the HIV-infected population has a diverse ethnicity and is, on average, younger [15]. It also has been pointed out that the 10-year risk model may underestimate the lifetime risk [35]. However, Framingham risk score has been largely validated against risk detected in the individual data of cohort studies [36], and its simplicity matches the aims of prevention [37]. 

Besides that, other limitations should be taken into account when interpreting the results. The use of the Framingham score may not capture the true risk for the HIV-infected population, and a specific score, taking into account the use of specific antiretroviral agents, might work differently [16]. In addition, the number of possible combinations of ARV agents and the duration of treatment makes it difficult to isolate individual effects on the risk of coronary heart disease. 

In conclusion, this study showed that cardiovascular risk factors are present in the HIV-infected population and account for risk of coronary heart disease in 10 years. Even with the variation on risk factor prevalence among regions, the main risk factors—hypertension and diabetes mellitus—were identified in all settings as risk factors for coronary heart disease. 

## Figures and Tables

**Table 1:** Characteristics of HIV-infected patients from regions of Brazil [*n* (% or mean ± SD)].

	Total (*n* = 3829)	Northeast (*n* = 2016)	Midwestern (*n* = 289)	Southern (*n* = 1224)	*P* value
Men	2086 (59.1)	1249 (62.0)	221 (76.5)	616 (50.3)	<0.001
Age (years)	39.0 ± 9.9	39.5 ± 9.6	37.3 ± 11.0	38.6 ± 10.1	<0.001
Nonwhite skin color	1194 (33.8)	529 (26.2)	145 (50.2)	520 (42.5)	<0.001
Years at school ≥10	1419 (40.2)	846 (42.0)	172 (59.5)	401 (32.8)	<0.001
Lifetime smoking	1424 (40.4)	480 (23.8)	137 (47.4)	807 (65.9)	<0.001
Physical activity	1171 (33.2)	389 (19.3)	74 (25.6)	708 (57.8)	<0.001
Binge drinking	747 (21.2)	475 (23.6)	66 (22.8)	206 (16.8)	<0.001
Current or past cocaine use	618 (17.5)	220 (10.9)	33 (11.6)	365 (29.8)	<0.001
Body mass index (kg/m^2^)					<0.001
≤25.0	2240 (63.9)	1357 (67.3)	184 (68.9)	699 (57.1)	
25.0–29.9	961 (27.4)	517 (25.6)	69 (25.8)	375 (30.6)	
≥30.0	306 (8.7)	142 (7.0)	14 (5.2)	150 (12.3)	
Waist circumference ≥102/88 cm	666 (19.0)	379 (18.8)	31 (11.6)	256 (20.9)	0.002
Hypertension status					<0.001
Normal	1406 (39.8)	721 (35.8)	102 (35.3)	583 (47.6)	
Prehypertension	1107 (31.4)	574 (28.5)	129 (44.6)	404 (33.0)	
Hypertension	1016 (28.8)	721 (35.8)	58 (20.1)	237 (19.4)	
Diabetes mellitus	473 (13.4)	364 (18.1)	10 (3.5)	99 (8.1)	<0.001
Antiretroviral use	2544 (72.1)	1544 (76.6)	192 (66.4)	808 (66.0)	<0.001
Time >8 years since HIV diagnosis	864 (24.5)	553 (27.4)	39 (13.5)	272 (22.2)	<0.001

**Table 2:** Association of risk factors with intermediate and high Framingham risk score among HIV-infected patients by regions of Brazil (*n* (%)).

	Northeast (*n* = 2016)	Midwestern (*n* = 289)	Southern (*n* = 1224)
Sex			
Men	24 (1.9)	3 (1.4)	18 (2.9)
Women	55 (7.2)	9 (13.2)	37 (6.1)
*P* value	<0.001	<0.001	0.008
Age (years)			
<50	20 (1.2)	1 (0.4)	9 (0.9)
50–59	42 (16.9)	7 (26.9)	24 (17.9)
≥60	17 (35.4)	4 (40.0)	22 (55.0)
*P* value	<0.001	<0.001	<0.001
Skin color			
Nonwhite	28 (5.3)	4 (2.8)	18 (3.5)
White	51 (3.4)	8 (5.6)	37 (5.3)
*P* value	0.06	0.2	0.13
Years at school			
<10	53 (4.5)	8 (6.8)	38 (4.6)
≥10	26 (3.1)	4 (2.3)	17 (4.2)
*P* value	0.1	0.06	0.8
Lifetime smoking			
No	58 (3.8)	4 (2.6)	14 (3.4)
Yes	21 (4.4)	8 (5.8)	41 (5.1)
*P* value	0.6	0.17	0.17
Physical activity			
No	64 (3.9)	9 (4.2)	25 (4.8)
Yes	15 (3.9)	3 (4.1)	30 (4.2)
*P* value	0.9	0.9	0.6
Binge drinking			
No	72 (4.7)	7 (3.1)	50 (4.9)
Yes	7 (1.5)	5 (7.6)	5 (2.4)
*P* value	0.002	0.11	0.12
Current or past cocaine use			
No	77 (4.3)	10 (4.0)	50 (5.8)
Yes	2 (0.9)	1 (3.0)	5 (1.4)
*P* value	0.02	0.8	<0.001
Body mass index (kg/m^2^)			
≤25.0	45 (3.3)	4 (2.2)	17 (2.4)
25.0–29.9	20 (3.9)	6 (8.7)	25 (6.7)
≥30.0	14 (9.9)	1 (7.1)	13 (8.7)
*P* value	0.001	0.06	<0.001
Waist circumference (cm)			
<102/88	46 (2.8)	5 (2.1)	27 (2.8)
≥102/88	33 (8.7)	6 (19.4)	28 (10.9)
*P* value	<0.001	<0.001	<0.001
Hypertension status			
Normal	12 (1.7)	0	3 (0.5)
Prehypertension	11 (1.9)	3 (2.3)	15 (3.7)
Hypertension	56 (7.8)	9 (15.5)	37 (15.6)
*P* value	<0.001	<0.001	<0.001
Diabetes mellitus			
No	25 (1.5)	6 (2.2)	32 (2.8)
Yes	54 (14.8)	6 (60.0)	23 (23.2)
*P* value	<0.001	<0.001	<0.001
Antiretroviral use			
No	22 (4.7)	2 (2.1)	14 (3.4)
Yes	57 (3.7)	10 (5.2)	41 (5.1)
*P* value	0.3	0.2	0.17
Time since HIV diagnosis (years)			
8–29	27 (34.2)	3 (25.0)	7 (12.7)
<8	52 (65.8)	9 (75.0)	48 (87.3)
*P* value	0.17	0.2	0.08

**Table 3:** Risk factors for intermediate and Framingham risk score among HIV-infected patients from Brazil.

	RR (95% CI)	*P* value
Study site in Brazil*		0.12
Northeast	1.0	
Midwestern	0.8 (0.4–1.4)	
Southern	0.7 (0.5–0.9)	
Sex*		<0.001
Men	1.0	
Women	3.0 (2.1–4.2)	
Age (years)*		<0.001
<50	1.0	
50–59	16.3 (10.8–24.7)	
≥60	43.4 (28.5–66.0)	
Skin color*		0.7
Nonwhite	1.0	
White	1.1 (0.8–1.4)	
Years at school*		0.9
<10	1.0	
≥10	1.0 (0.7–1.4)	
Lifetime smoking*		<0.001
No	1.0	
Yes	2.2 (1.6–3.0)	
Physical activity*		0.9
No	1.0	
Yes	1.0 (0.7–1.4)	
Binge drinking*		0.04
No	1.0	
Yes	0.6 (0.4–1.0)	
Current or past cocaine use*		0.13
No	1.0	
Yes	0.6 (0.3–1.2)	
Body mass index (kg/m^2^)*		<0.001
≤25.0	1.0	
25.0–29.9	1.3 (0.9–1.8)	
≥30.0	2.3 (1.5–3.4)	
Waist circumference (cm)*		0.001
<102/88	1.0	
≥102/88	1.7 (1.3–2.4)	
Hypertension status**		<0.001
Normal	1.0	
Prehypertension	2.1 (1.2–3.6)	
Hypertension	4.6 (2.7–7.9)	
Diabetes mellitus**		<0.001
No	1.0	
Yes	4.7 (3.4–6.5)	
Antiretroviral use*		0.7
No	1.0	
Yes	0.9 (0.7–1.3)	
Time since HIV diagnosis (years)*		0.6
<8	1.0	
8–29	0.8 (0.4–1.4)	

*Risk ratio was adjusted for study site, sex, age, years at school, smoking, binge drinking, cocaine use, antiretroviral use, and length of the HIV diagnosis.

**Risk ratio additionally adjusted for waist circumference, hypertension, and diabetes mellitus.
